# Classification of “Athlete's Heart” using machine learning of conventional 12-lead ECG: male elite 3,000-m runner data in the CHIEF study

**DOI:** 10.3389/fspor.2026.1847495

**Published:** 2026-06-17

**Authors:** Chia-Hao Fan, Chin-Fen Chen, Wei-Chun Huang, Younghoon Kwon, Xuemei Sui, Carl J. Lavie, Gen-Min Lin

**Affiliations:** 1 Department of Nursing, Hualien Tzu Chi Hospital, Buddhist Tzu Chi Medical Foundation, Hualien, Taiwan; 2 Department of Medicine, Hualien Armed Forces General Hospital, Hualien, Taiwan; 3 Department of Business Management, National Sun Yat-sen University, Kaohsiung City, Taiwan; 4 Department of Medicine, Pingtung Christian Hospital, Pingtung City, Pingtung, Taiwan; 5 Division of Cardiology, University of Washington, Harborview Medical Center, Seattle, WA, United States; 6 Department of Exercise Science, Arnold School of Public Health, University of South Carolina, Columbia, SC, United States; 7 John Ochsner Heart and Vascular Institute, Ochsner Clinical School, The University of Queensland School of Medicine, New Orleans, LA, United States; 8 Department of Medicine, Tri-Service General Hospital, National Defense Medical University, Taipei, Taiwan

**Keywords:** electrocardiographic, elite runner, machine learning, military personnel, Taiwanese

## Abstract

**Background:**

Conventional electrocardiographic (ECG) interpretive algorithms often struggle to distinguish physiological cardiovascular adaptations in athletes from cardiac pathology. This study utilized machine learning (ML) to estimate the likelihood of elite 3,000-meter running performance using resting ECG and biological markers within a large military cohort.

**Methods:**

We analyzed data from 2,296 physically active military males in the Cardiorespiratory Fitness and Health in Eastern Armed Forces (CHIEF) Heart Study. Three ML classifiers—logistic regression (LR), multilayer perceptron (MLP), and support vector machine (SVM)—were trained using 26 ECG features (axis, duration and voltage of P, QRS and T waves in each lead and supine heart rate) and 6 biological features (age, body weight, body height, waist circumference, blood pressure and sitting pulse rate) to classify “elite” runners (defined as the top 5% and 10% performance groups). The whole data were randomly grouped by a 3:1 ratio into a training/validation set (*N* = 1,607) and a test set (*N* = 689).

**Results:**

For the top 10% runners, LR demonstrated the highest discriminative power (AUC: 80.81%), followed by MLP (78.43%). In the top 5% group, MLP performed best with an AUC of 74.42%. When specificity was fixed at approximately 60%–70%, the sensitivity of the optimal models for both groups exceeded 81%.

**Conclusions:**

ML can effectively classify elite endurance capacity using non-invasive resting ECG markers. These findings highlight the potential for updating automated ECG interpretive algorithms to better recognize “athlete's heart.” Furthermore, this approach may serve as a cost-effective preliminary screening tool for identifying elite athletic potential, although further validation in female and more diverse populations is warranted.

## Introduction

The physiological adaptation of the heart to chronic, intensive physical training is commonly referred to as the “athlete's heart” (1–5). These adaptations, characterized by structural remodeling, such as chamber enlargement and myocardial hypertrophy, are essential for sustaining high cardiac output during endurance exercise (2). On a standard 12-lead electrocardiogram (ECG), these changes often manifest as distinct electrical signatures, including increased voltage of the QRS complex, early repolarization patterns, and various forms of sinus bradycardia or conduction delays (3). While these markers are generally considered benign physiological responses, they reflect the profound efficiency of the cardiovascular (CV) system in elite performers (1, 4). However, distinguishing these physiological adaptations from pathological conditions remains a clinical challenge, as current automated ECG interpretive algorithms frequently lack the refinement to accurately differentiate “athlete's heart” from pathologies like hypertrophic cardiomyopathy.

The 3,000-meter run is a widely recognized benchmark for evaluating aerobic capacity and cardiorespiratory fitness (CRF), particularly in military and athletic populations (6, 7). Within the context of the CHIEF study, our previous research has delineated the specific prevalence and ECG manifestations of the “athlete's heart” among Asian military males, providing a critical foundation for using ECG to assess physical fitness in this demographic (8). Traditionally, identifying individuals with superior running potential relied on direct physical assessment or professional coaching intuition (9). Although ECG features have been identified as potential indicators of athletic performance, traditional diagnostic criteria often focus on single-variable thresholds (e.g., Sokolow-Lyon criteria for hypertrophy) (5, 10). These conventional methods frequently fail to account for the complex, non-linear interactions between multiple electrical parameters and biological variables—such as age, body mass index (BMI), and resting blood pressure and pulse rate—which collectively define the elite athletic phenotype.

In recent years, the integration of artificial intelligence (AI) and machine learning (ML) has revolutionized clinical diagnostics by enabling the analysis of high-dimensional data (11–16). ML algorithms, such as Logistic Regression (LR), Multilayer Perceptron (MLP), and Support Vector Machines (SVM), are particularly adept at recognizing subtle patterns within large datasets that may be invisible to the human eye or overlooked by standard statistical models. Despite the growing use of ML in detecting cardiac pathologies, its application in classifying high-performance traits, specifically elite endurance capacity, remains relatively unexplored. Notably, improving these automated interpretive capabilities could significantly assist clinicians who currently rely heavily on standard machine interpretations (17).

Therefore, the present study sought to bridge this gap by using the CHIEF Heart Study. We aimed to develop and validate various ML classifiers to estimate the likelihood of elite 3,000-m runners among a large population of physically active military males. By integrating 26 ECG-derived features with 6 essential biological characteristics, we hypothesized that an ML approach could provide a more accurate and automated method for classifying superior exercise performance compared to traditional measures, potentially informing future updates to automated ECG interpretive algorithms.

## Methods

### Study population

A population of 2,296 military males, aged 17–43 years, were obtained from the CHIEF Heart Study to evaluate the efficacy of ML in classifying elite physical performance. All participants underwent an annual health examination, which included demographic, anthropometric, and hemodynamic measurements at the Hualien Armed Forces General Hospital in Taiwan from 2016 to 2021. Physical performance was assessed using a 3,000-meter run test. This study was approved by the Institutional Review Board of the Tri-Service General Hospital (TSGHIRB No.： A202505143) in Taipei City, Taiwan and written informed consent was obtained from all participants.

The term “elite athlete” is commonly discussed in sports science but lacks a precise, universal definition. For the purpose of this study, participants were categorized into two groups based on their performance:

(1) Elite Group is respectively defined as the top 5% and 10% of performers, and (2) Control Group is respectively defined as the remaining 95% and 90% of the population.

### Clinical and ECG measurements

Anthropometric parameters, i.e., body height, body weight and waist circumference (WC) were measured in a standing position. Hemodynamic parameters, specifically systolic and diastolic blood pressure (SBP/DBP) and resting pulse rate, were measured after at least 15 min of rest using an automatic oscillometric monitor.

A 12-lead ECG was performed for each participant in a supine position (Schiller AG CARDIOVIT MS-2015, Baar, Switzerland). To ensure data integrity, any ECG report with significant technical artifacts—defined as non-physiological interference such as baseline wandering, electrode motion, or muscle tremors—was repeated by a professional technician. Standard 10-second 12-lead ECG recordings were re-acquired until a noise-free tracing was obtained for analysis. The analysis for the ECG parameters such as resting heart rate (RHR) and P-QRS-T wave duration or interval was performed by the built-in software of the ECG machine and confirmed by a board-certified cardiologist. Notably, while both RHR (from ECG) and sitting pulse rate (from the oscillometric monitor) were recorded, RHR was prioritized in our primary analysis due to the higher precision of ECG-based calculations.

### ML procedures

Three ML classifiers including the MLP, LR and SVM with a linear kernel were used for 26 ECG features (RHR; P wave duration in lead II; intervals of PR, QRS and QT in lead II; axes of P, QRS and T waves in lead II; voltages of the R wave in limb leads I, II, III, aVR, aVL and aVF; voltages of both the R and S waves in precordial leads V1–V6), and with or without 6 biological features (age, body height, body weight, WC, SBP, DBP and sitting pulse rate) training to identify the elite runners. To ensure uniform contribution of features to the models, Min-Max scaling was applied to normalize all input data into a linear transformation range between 0 and 1.

### Data augmentation and cross validation

**Figure 1:**
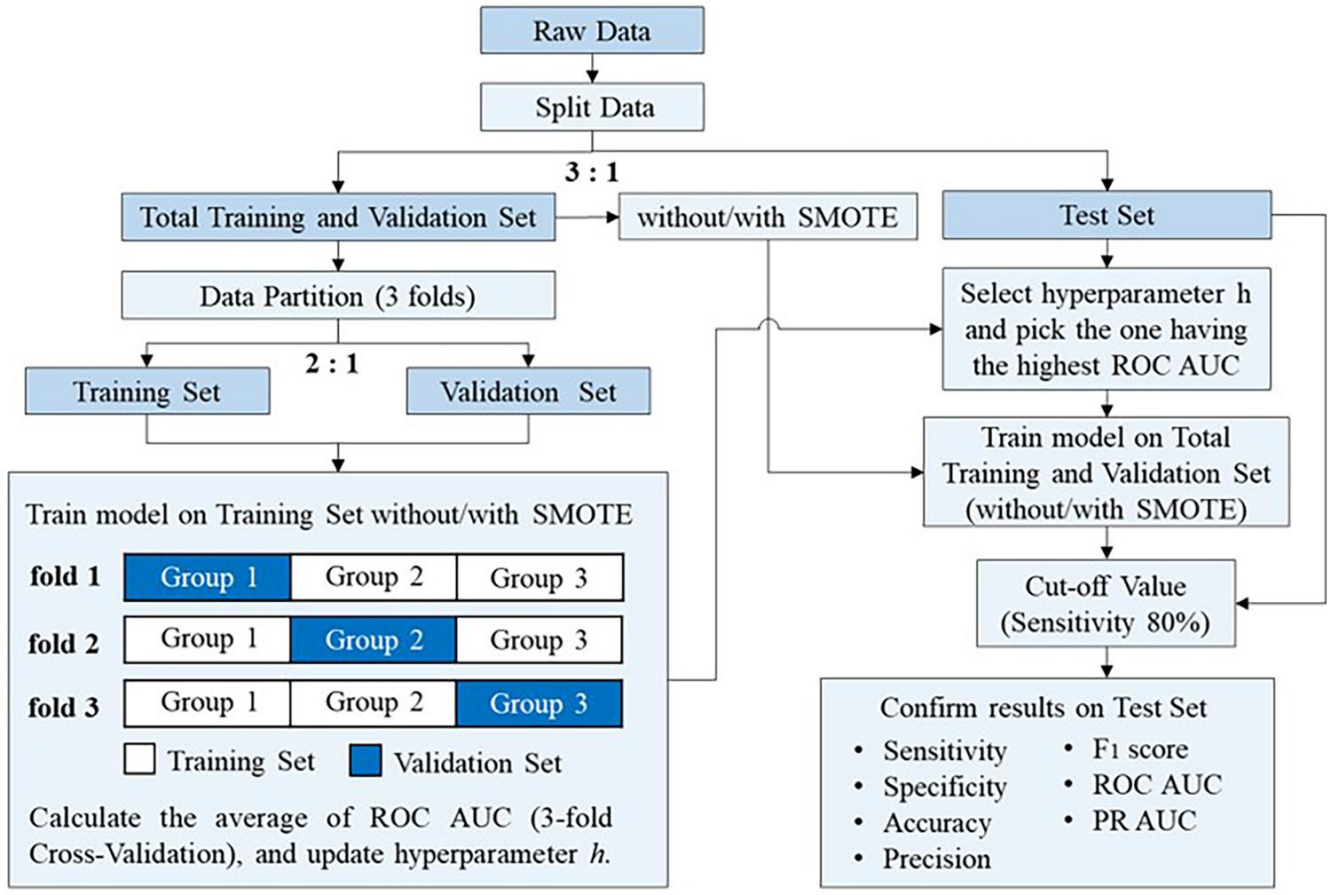
The flowchart of the proposed method.

The dataset of 2,296 participants was randomly partitioned into a training/validation set (*N* = 1,607) and an independent test set (*N* = 689) using a 3:1 ratio. To address the inherent class imbalance, where elite runners represented only small percentages of the population (5% or 10%), the Synthetic Minority Over-sampling Technique (SMOTE) (18) was applied. This technique was integrated into the 4-fold cross-validation process to augment the training data of the elite group to match the size of the control group (Figure 1). The specific distribution of participants and the number of samples in each fold of the cross-validation process are detailed in Table 1.

**Table 1:** Data numbers in the dataset.

Fold	Data	3,000 m Except	3,000 m	3,000 m Except	3,000 m	Total
		Top 5%	Top 5%	Top 10%	Top 10%	
1st	Training set	1,023	48	967	104	1,071
	Pre-processed by SMOTE	1,023	1,023	967	967	2,046/1,934
	Validation set	508	28	479	57	536
2nd	Training set	1,017	54	963	108	1,071
	Pre-processed by SMOTE	1,017	1,017	963	963	2,034/1,926
	Validation set	514	22	483	53	536
3rd	Training set	1,022	50	962	110	1,072
	Pre-processed by SMOTE	1,022	1,022	962	962	2,044/1,924
	Validation set	509	26	484	51	535
–	Testing set	652	37	623	66	689

The performance of the three classifiers was evaluated using the Area Under the Receiver Operating Characteristic (ROC) curve (AUC). To assess clinical utility, the sensitivity of the models was estimated by fixing the specificity at approximately 70.0%. Statistical comparisons were performed using the scikit-learn v0.20.2 library and Python programming language.

## Results

### Participant characteristics

The baseline characteristics of the study population are summarized in Table 2. A total of 2,296 military males were analyzed, with “Elite” 3,000-m runners (*N* = 227) using the cut-off level of 10% in Table 1. Significant physiological differences were observed between the elite group and the control group. Elite runners were significantly younger (26.80 ± 5.74 vs. 29.25 ± 5.66 years, *p* < 0.0001), had lower body weight (68.35 ± 8.58 vs. 73.45 ± 10.18 kg, *p* < 0.0001), and smaller WC (77.89 ± 9.94 vs. 82.77 ± 10.54 cm, *p* < 0.0001).

**Table 2:** Characteristics of study participants.

Features	Total	3,000 m Except top 10% (control group)	3,000 m top 10% (elite group)	*p*-value
	*N* = 2,296	*N* = 2,069	*N* = 227	
Age (years)	29.01 ± 5.71	29.25 ± 5.66	26.80 ± 5.74	<0.0001
Height (cm)	171.72 ± 5.77	171.75 ± 5.84	171.44 ± 5.17	0.4056
Weight (kg)	72.95 ± 10.15	73.45 ± 10.18	68.35 ± 8.58	<0.0001
Waist (cm)	82.28 ± 10.58	82.77 ± 10.54	77.89 ± 9.94	<0.0001
Heart rate (bpm)	62.72 ± 9.31	63.08 ± 9.16	59.45 ± 10.02	<0.0001
Pulse rate (bpm)	71.67 ± 10.56	72.15 ± 10.49	67.26 ± 10.28	<0.0001
PR-II (ms)	161.62 ± 22.46	161.60 ± 22.31	161.85 ± 23.76	0.8750
P-II (ms)	98.93 ± 21.14	98.82 ± 21.04	99.88 ± 22.05	0.4738
QRS-II (ms)	95.77 ± 10.65	95.57 ± 10.55	97.58 ± 11.38	0.0070
QTc-II (ms)	383.73 ± 21.10	383.83 ± 21.25	382.82 ± 19.66	0.4940
P axis-II (degree)	45.79 ± 27.86	46.03 ± 27.53	43.62 ± 30.70	0.2573
QRS axis-II (degree)	64.80 ± 32.43	64.01 ± 32.43	72.00 ± 31.58	0.0004
T axis-II (degree)	39.31 ± 19.55	38.90 ± 19.64	43.04 ± 18.29	0.0025
R-I (mm)	5.22 ± 2.70	5.27 ± 2.70	4.79 ± 2.66	0.0121
R-II (mm)	10.89 ± 3.96	10.74 ± 3.92	12.29 ± 4.08	<0.0001
R-III (mm)	7.47 ± 4.62	7.26 ± 4.53	9.42 ± 4.94	<0.0001
R-aVR (mm)	0.74 ± 0.26	0.73 ± 0.26	0.78 ± 0.27	0.0050
R-aVL (mm)	2.36 ± 2.31	2.39 ± 2.28	2.04 ± 2.52	0.0421
R-aVF (mm)	8.67 ± 4.12	8.51 ± 4.05	10.13 ± 4.44	<0.0001
R-V1 (mm)	3.30 ± 2.39	3.28 ± 2.36	3.55 ± 2.64	0.1359
S-V1 (mm)	9.42 ± 4.50	9.27 ± 4.41	10.79 ± 5.02	<0.0001
R-V2 (mm)	6.95 ± 3.53	6.95 ± 3.55	6.91 ± 3.35	0.8465
S-V2 (mm)	14.76 ± 5.93	14.64 ± 5.88	15.94 ± 6.28	0.0017
R-V3 (mm)	10.74 ± 4.84	10.69 ± 4.79	11.17 ± 5.31	0.1883
S-V3 (mm)	8.83 ± 5.31	8.81 ± 5.30	9.00 ± 5.37	0.6266
R-V4 (mm)	17.07 ± 5.25	16.96 ± 5.23	18.09 ± 5.33	0.0021
S-V4 (mm)	4.39 ± 4.04	4.45 ± 4.10	3.86 ± 3.42	0.0159
R-V5 (mm)	16.80 ± 4.84	16.72 ± 4.79	17.52 ± 5.16	0.0172
S-V5 (mm)	2.26 ± 2.65	2.29 ± 2.67	1.98 ± 2.42	0.0922
R-V6 (mm)	13.49 ± 4.11	13.47 ± 4.12	13.72 ± 4.03	0.3723
S-V6 (mm)	1.07 ± 2.16	1.08 ± 2.21	0.90 ± 1.63	0.1193
3,000 m (s)	859.05 ± 72.76	871.29 ± 65.41	747.46 ± 29.23	<0.0001

Regarding hemodynamic and ECG parameters, elite runners exhibited a significantly lower RHR (59.45 ± 10.02 vs. 63.08 ± 9.16 bpm, *p* < 0.0001) and sitting pulse rate. Several ECG morphology features, particularly voltage-related metrics in the precordial leads, showed distinct patterns in the elite group, reflecting the typical electrical remodeling of an “athlete's heart.”

### ML model performance

**Figure 2:**
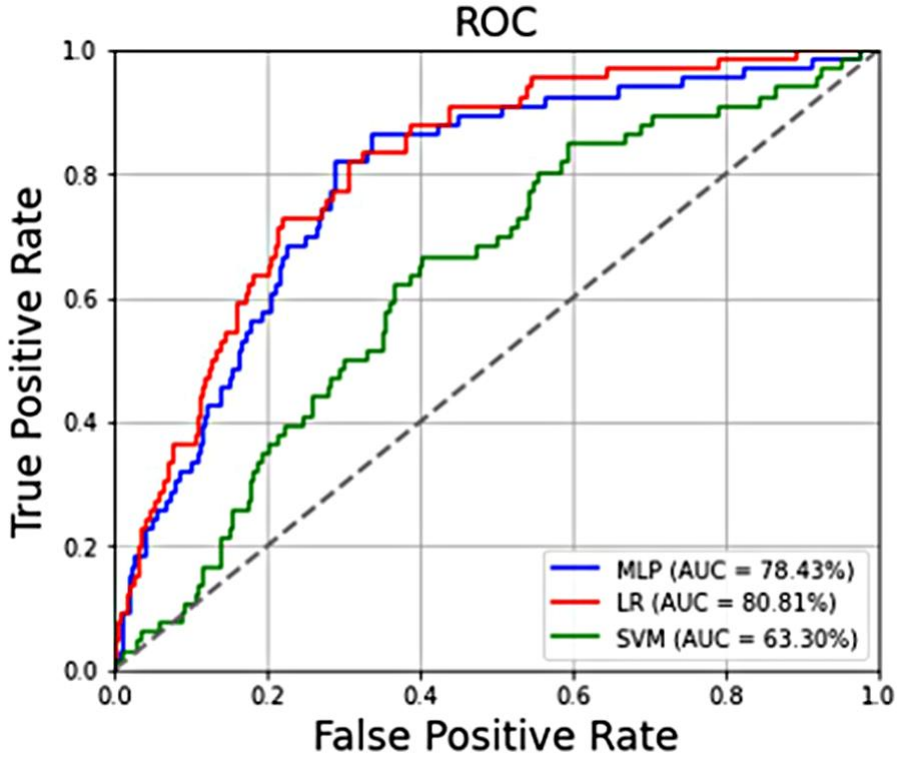
Receiver operating characteristic curves and precision recall curves (top 10% without SMOTE).

The performance of the three ML classifiers, which were configured with the optimal hyperparameters listed in Table 3, is illustrated by the Receiver ROC curves in Figure 2. Among the models tested, LR demonstrated the highest predictive accuracy with an AUC of 80.81%, closely followed by the MLP, which achieved an AUC of 78.43%. In contrast, the SVM yielded a significantly lower AUC of 63.30%. A comprehensive comparison of the performance metrics for all machine learning models, including sensitivity, specificity, and F1 scores for both the top 5% and top 10% runner groups, is presented in Table 4.

**Table 3:** Hyperparameter optimization (top 10%).

Model	Hyperparameter	Beginning value	Ending value	Interval	Optimal value	
					Without SMOTE	With SMOTE
Support vector machine	Regularization	0.01	1.00	0.01	0.29	0.11
Logistic regression	Regularization	0.01	10.00	0.01	0.94	0.23
Multilayer perceptron	Regularization	0.10	10.00	0.10	1.00	3.40
	Number of hidden layers	–	–	–	3	3
	Number of neurons	–	–	–	20, 10, 5	20, 10, 5
	Number of iterations	–	–	–	10,000	10,000

The hyperparameters that are not described in this table are set to the default values used in the Scikit-learn library.

**Table 4:** Performance comparison of proposed method and previous work.

The run performance	Preprocessing	Machine learning classifiers	Sensitivity	Specificity	Accuracy	Precision	*F*_1_ score	ROC AUC	PR AUC
Top 5%	Without SMOTE	Multilayer perceptron	81.08%	60.89%	61.97%	10.53%	18.63%	74.42%	11.12%
		Logistic regression	81.08%	48.47%	50.22%	8.20%	14.89%	72.11%	11.02%
		Support vector machine	81.08%	24.85%	27.87%	5.77%	10.77%	55.92%	6.89%
	With SMOTE	Multilayer perceptron	81.08%	45.55%	47.46%	7.79%	14.22%	71.02%	10.75%
		Logistic regression	81.08%	49.54%	51.23%	8.36%	15.15%	69.04%	9.42%
		Support vector machine	81.08%	44.02%	46.01%	7.59%	13.89%	71.40%	10.78%
Top 10%	Without SMOTE	Multilayer perceptron	81.82%	70.95%	71.99%	22.98%	35.88%	78.43%	25.41%
		Logistic regression	81.82%	69.18%	70.39%	21.95%	34.62%	80.81%	28.97%
		Support vector machine	81.82%	41.57%	45.43%	12.92%	22.31%	63.30%	13.29%
	With SMOTE	Multilayer perceptron	81.82%	65.65%	67.20%	20.15%	32.34%	79.33%	29.24%
		Logistic regression	80.30%	65.65%	67.05%	19.85%	31.83%	79.15%	28.01%
		Support vector machine	80.30%	67.42%	68.65%	20.70%	32.92%	79.08%	27.64%

When specificity was fixed at approximately 70.0%, both the LR and MLP models achieved a high sensitivity of 81.82% in identifying elite runners in the test set. These results suggest that the combination of 26 ECG features and 6 biological variables contain sufficient signal for the LR and MLP algorithms to effectively distinguish top-tier aerobic performance (Figure 2). Notably, while SMOTE preprocessing significantly improved the performance of the SVM, it did not lead to substantial improvements for the MLP and LR models in predicting either top 5% or top 10% runners.

### Feature importance analysis

**Figure 3:**
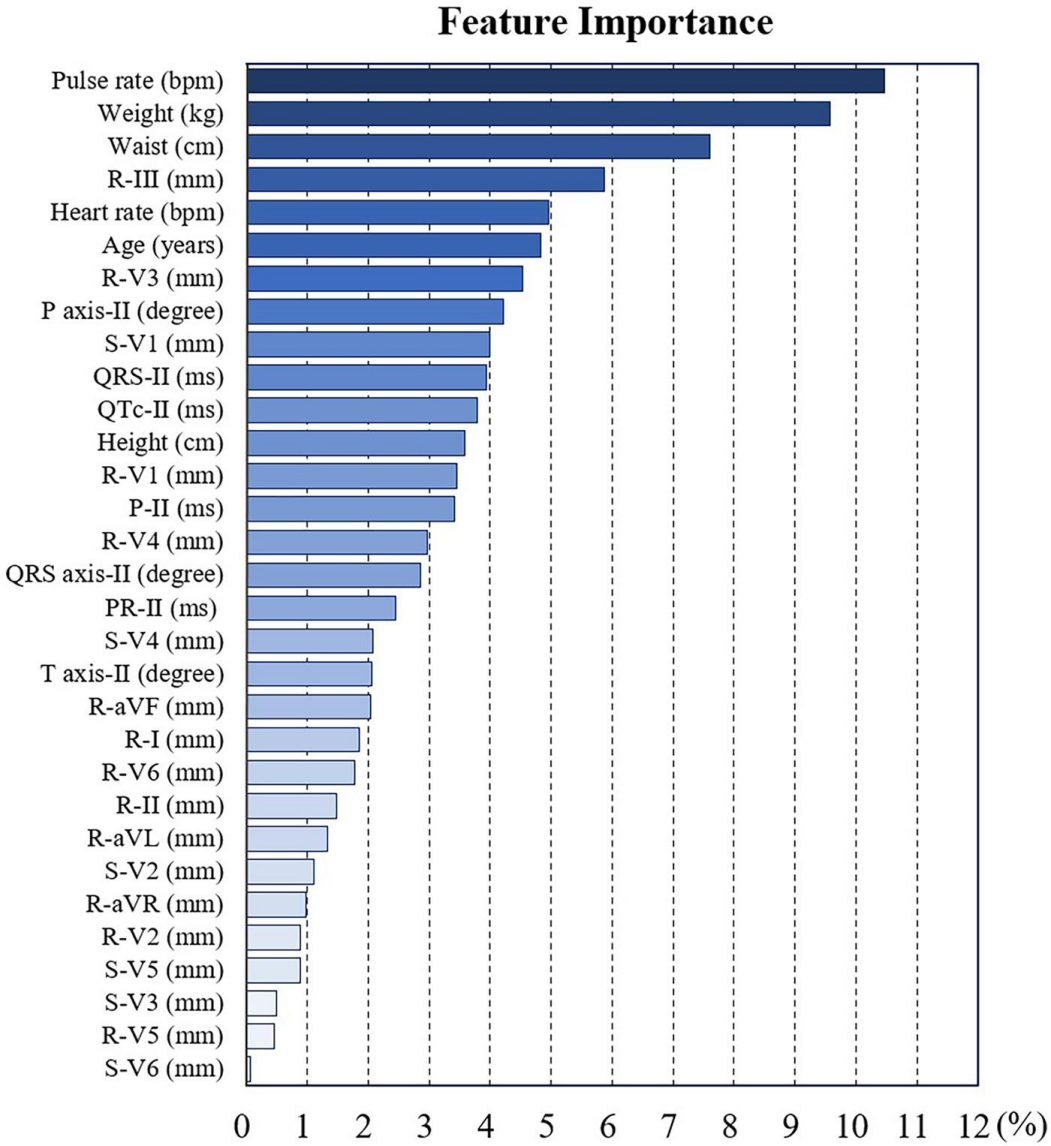
Feature importance—logistic regression.

Because the ML models demonstrated higher discriminative performance for the top 10% elite runners compared to the top 5% group, the Feature Importance Analysis (FIA) focused on the 10% classification model. The FIA, derived from the best-performing Logistic Regression (LR) model, revealed the primary drivers of the classification. As illustrated in the importance ranking (Figure 3), sitting pulse rate and body weight were the two most influential biological features for estimating the likelihood of elite status.

Furthermore, several ECG-specific markers played a critical role in the model's decision-making process. Specifically, the R-wave voltage in Lead III (R-III), resting heart rate (RHR), and P-wave axis in Lead II (P-axis-II) were among the top-ranked features. The significant contribution of voltage-based features (e.g., R-V3 and S-V1) suggests that ventricular voltage criteria—traditionally used to diagnose left ventricular hypertrophy—also serve as robust indicators of the superior cardiac output and structural adaptations found in elite 3,000-m runners. The prominence of both sitting pulse rate and RHR as high-ranking features emphasizes the importance of autonomic efficiency in athletic performance, though their distinct rankings may reflect differences in measurement precision or physiological state during data collection.

## Discussion

**Figure 4:**
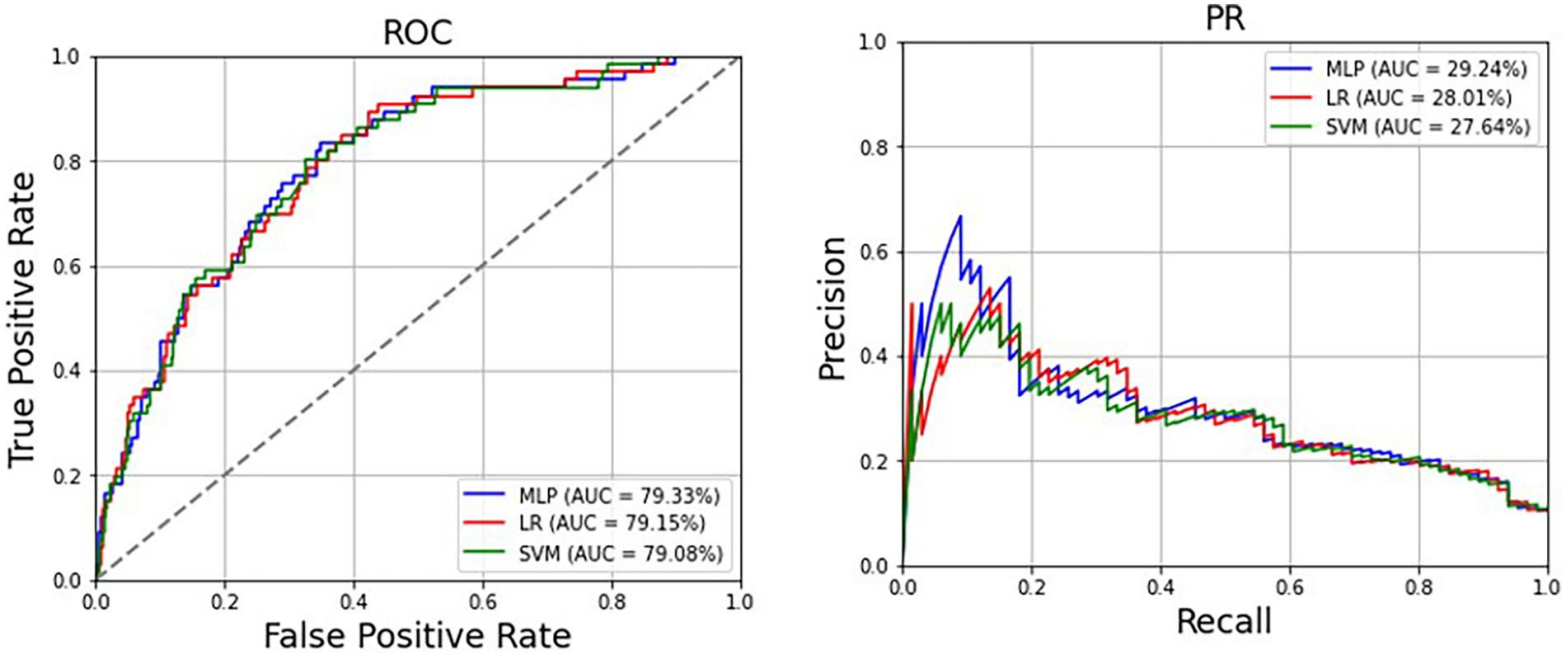
Receiver operating characteristic curves and precision recall curves (top 10% with SMOTE).

To the best of our knowledge, this is the first study to utilize ML algorithms for the integration of ECG and biological features to specifically classify elite 3,000-meter running performance within a large-scale military population. While previous research has extensively characterized “athlete's heart” through manual ECG interpretation (19, 20), our study pioneeringly demonstrates that automated ML classifiers—specifically MLP and LR—can capture subtle electrical and physiological signatures to estimate the likelihood of top-tier aerobic endurance performers (top 5% and 10%) with high accuracy. Furthermore, while SMOTE preprocessing showed limited impact on LR and MLP, it significantly improved SVM performance, enabling it to achieve discriminative levels comparable to the other models (Figure 4).

The emergence of RHR and body weight as critical predictors aligns with established physiological principles. Elite endurance capacity is fundamentally linked to increased vagal tone and stroke volume, which manifest as resting bradycardia (21, 22). Our model's reliance on pulse rate reinforces the notion that the efficiency of the autonomic nervous system is a primary determinant of aerobic success.

Regarding ECG features, the high importance of voltage-related features (e.g., R-III, R-V3, and S-V1) reflects the electrical remodeling characteristic of the “athlete's heart.” In accordance with the International Recommendations for Electrocardiographic Interpretation in Athletes (23), increased QRS voltage is a common physiological adaptation in endurance athletes due to LVH. Our study extends this understanding by showing that these electrical markers are not merely signs of adaptation but are powerful, quantifiable predictors of functional performance. Unlike traditional manual interpretation, which often classifies these markers as “borderline” or “normal variants,” our ML approach treats them as high-fidelity signals of superior CV output.

Regarding practical deployment, the transition from research-oriented classification to clinical decision-making requires rigorous probability calibration. While our models demonstrate acceptable discriminative power, future work should involve reliability diagrams and Brier score evaluations to ensure that predicted probabilities align with actual observed frequencies. Furthermore, the selection of optimal thresholds (e.g., via Youden's Index) must be tailored to the specific context—prioritizing high sensitivity for preliminary screening in elite sports recruitment vs. high specificity to avoid unnecessary diagnostic cascades in clinical settings.

Historically, classifying elite athletes has relied on expensive and labor-intensive tests, such as maximal oxygen consumption (VO_2_max) on treadmill protocols (24, 25). This study offers a lower-cost, scalable alternative. The superior performance of LR and MLP over SVM suggests a robust, largely linear relationship between the integrated features and athletic performance. By achieving an AUC of 80.81%, our LR model demonstrates that the “signal” for elite performance is deeply embedded within the 32 variables used. The utility of data-driven approaches in running physiology extends beyond ECG markers. For instance, recent studies have demonstrated the efficacy of interpretable ML in identifying biomechanical imbalances, such as hamstrings-quadriceps discrepancies, using simulation-derived features. Integrating such multi-dimensional data—ranging from cardiovascular electrical signatures to musculoskeletal biomechanics—could further refine the conceptual framing of athletic performance assessment (26). While Corrado et al. (2008) focused on ECG for sudden cardiac death prevention (27), our research pivots the utility of ECG toward performance phenotyping, filling a significant gap in automated physical training monitoring.

### Study limitations

Several limitations of this study warrant acknowledgment. First, the study population consisted exclusively of young, physically active Taiwanese military males. Therefore, the generalizability of our findings to females, older populations, or different ethnic groups remains to be established. We also acknowledge that age is a potential confounding factor regarding athletic cardiac adaptation. While younger athletes often exhibit Respiratory Sinus Arrhythmia (RSA) as a sign of healthy vagal tone, older populations may demonstrate increased ectopic activity or atrial fibrillation; future research should explicitly account for these age-dependent rhythmic variations when training ML models.

Second, while the integration of 32 features provided high discriminative power, the presence of potentially redundant variables—such as both sitting pulse rate and ECG-derived RHR—may introduce multicollinearity. Future iterations of the model should prioritize ECG-derived RHR to ensure measurement consistency and physiological accuracy, as oscillometric pulse rate may be susceptible to minor measurement artifacts. Furthermore, future modeling should consider integrating more refined hemodynamic parameters. Specifically, Mean Arterial Pressure (MAP) could be incorporated as a key feature in reanalysis or future prospective studies. As MAP represents the average arterial pressure during a single cardiac cycle, its inclusion may provide a more stable and comprehensive indicator of cardiovascular workload and peripheral perfusion compared to systolic or diastolic pressures alone in elite athletes.

Third, although SMOTE was utilized to address class imbalance, it primarily benefited the SVM model, suggesting that the underlying physiological “signal” of elite performance in this dataset is inherently more compatible with linear or network-based architectures like LR and MLP. Finally, this study relied on a 3,000-m run as the sole metric for performance; incorporating additional markers, such as VO_2_ max or echocardiographic data, would further validate the structural correlates of the “athlete's heart.”

## Conclusion

This study is the first to demonstrate that ML can successfully bridge the gap between resting ECG data and elite middle-distance running performance. By leveraging LR and MLP models, we achieved acceptable sensitivity in classifying top-tier runners, establishing a novel, non-invasive framework for athletic potential assessment.

Our findings indicate that the “signal” for superior aerobic endurance is deeply embedded in a combination of ECG voltage criteria and biological features, such as body weight and resting heart rate. These markers are not merely physiological adaptations but serve as robust indicators of functional capacity. Furthermore, this research provides a foundation for future digital health systems aimed at refining automated ECG interpretive algorithms, ensuring a more accurate differentiation between the “athlete's heart” and pathological conditions. Such advancements could offer a cost-effective preliminary screening tool for objective personnel selection and physical fitness monitoring in both military and professional sports environments.

## Data Availability

The original contributions presented in the study are included in the article/Supplementary Material, further inquiries can be directed to the corresponding author.
